# Solid Electrolyte Membranes Based on Li_2_O–Al_2_O_3_–GeO_2_–SiO_2_–P_2_O_5_ Glasses for All-Solid State Batteries

**DOI:** 10.3390/membranes12121245

**Published:** 2022-12-08

**Authors:** Svetlana V. Pershina, Tamara A. Kuznetsova, Emma G. Vovkotrub, Semyon A. Belyakov, Elena S. Kuznetsova

**Affiliations:** Institute of High Temperature Electrochemistry of the Ural Branch of the RAS, 20 Akademicheskaya St., 620990 Ekaterinburg, Russia

**Keywords:** all-solid-state batteries, solid electrolyte membrane, glasses, glass-ceramics, crystallization kinetics, Li_1.5_Al_0.5_Ge_1.5_(PO_4_)_3_

## Abstract

Rechargeable Li-metal/Li-ion all-solid-state batteries due to their high safety levels and high energy densities are in great demand for different applications ranging from portable electronic devices to energy storage systems, especially for the production of electric vehicles. The Li_1.5_Al_0.5_Ge_1.5_(PO_4_)_3_ (LAGP) solid electrolyte remains highly attractive because of its high ionic conductivity at room temperature, and thermal stability and chemical compatibility with electrode materials. The possibility of LAGP production by the glass-ceramic method makes it possible to achieve higher total lithium-ion conductivity and a compact microstructure of the electrolyte membrane compared to the ceramic one. Therefore, the crystallization kinetics investigations of the initial glass are of great practical importance. The present study is devoted to the parent glasses for the production of Li_1.5+x_Al_0.5_Ge_1.5_Si_x_P_3−x_O_12_ glass-ceramics. The glass transition temperature *T_g_* is determined by DSC and dilatometry. It is found that *T_g_* decreases from 523.4 (*x* = 0) to 460 °C (*x* = 0.5). The thermal stability of glasses increases from 111.1 (*x* = 0) to 188.9 °C (*x* = 0.3). The crystallization activation energy of Si-doped glasses calculated by the Kissinger model is lower compared to that of Si-free glasses, so glass-ceramics can be produced at lower temperatures. The conductivity of the glasses increases with the growth of *x* content.

## 1. Introduction

Lithium-ion batteries are in demand in all spheres of human activity, from portable electronics to electric vehicles and spacecraft due to their high safety levels and high energy density [1,2,3]. Commercially produced lithium-ion batteries present an inherent hazard of liquid electrolyte leakage, and, when damaged, they are prone to swelling due to changes in temperature. Switching from liquid electrolytes to solid electrolyte membranes can decide the safety issues of lithium-ion power sources [3,4].

Among the numerous classes of oxide conductors reported in recent years, lithium-conducting glasses and glass-ceramics are the most promising solid electrolytes for all-solid-state batteries [2,5,6,7]. Moreover, similar glass-forming systems have a wider application both in optical materials and in nuclear technologies [8,9]. The Li_2_O–Al_2_O_3_–GeO_2_–P_2_O_5_ glass-forming system is of particular interest since it can be used as a basis for producing NASICON-structured glass-ceramic electrolytes of the Li_1+x_Al_x_Ge_2−x_(PO_4_)_3_ series, which have a high conductivity (10^−4^ S cm^−1^ at room temperature (RT)), thermal stability, compact microstructure, and chemical compatibility with electrode materials [7,10,11]. All-solid-state batteries with Li_1.5_Al_0.5_Ge_1.5_(PO_4_)_3_ (or LAGP) solid electrolyte (LiFePO_4_ cathode and Li anode) demonstrate a cycling capacity of 131.3 mAh g^−1^ after 1000 cycles and a high rate cycling stability of 75 mAh g^−1^ at 5 C, 50 °C [12].

It should be pointed out that the electrical properties of glass-ceramics are considerably dependent on the chemical composition and thermal history [11,13,14]. Thus, the conductivity of lithium-germanium-phosphate glass-ceramics increases with an increase in Al_2_O_3_ content from 2.25·10^−8^ S cm^−1^ (LiGe_2_(PO_4_)_3_ composition) to 5.03·10^−4^ S cm^−1^ (LAGP) at 25 °C [11]. In [14], the effect of the microstructure of the crystallized LAGP glass on the conductivity is discussed. Controlled glass crystallization result in the glass-ceramics with a homogenous microstructure, which leads to higher conductivity compared to ceramics of the same composition. In [15], the effect of the crystallization temperature on the conductivity of LAGP was studied, which increased from 1.61·10^−3^ S cm^−1^ to 2.91·10^−3^ S cm^−1^ at heat treatment temperatures of 750 and 800 °C, respectively. It has been found that to obtain highly conductive LAGP glass-ceramics with the dense microstructure, heat treatment is required at temperatures significantly higher than the crystallization peak temperature, since the activation energy for crystallization (*E_c_*) is quite high (~400 kJ mol^−1^) [10,11,16]. Crystallization kinetics is often studied using a non-isothermal model [17,18]. It has been established that doping Li_2_O–GeO_2_–P_2_O_5_ glass with Al_2_O_3_ leads to decrease in *E_c_* from 328 to 300 kJ mol^−1^ [10]. The *E_c_* of 20Li_2_O–6Al_2_O_3_–35GeO_2_–38P_2_O_5_ glass is reported to be 442 kJ mol^−1^ [19]. Previously, we demonstrated that Al_2_O_3_ facilitates the processes of glass crystallization and that *E_c_* obtained by the Kissinger model decreases from 435 to 400 kJ mol^−1^ for 12.5Li_2_O–50GeO_2_–37.5P_2_O_5_ and 20.63Li_2_O–8.12Al_2_O_3_–33.75GeO_2_–37.50P_2_O_5_ glasses, respectively [16]. It has also been found that both the glass transition temperature and the crystallization temperature decrease with the introduction of alumina. In addition, the lithium-ion conductivity was increased by 18 times compared to undoped glass.

Doping of LAGP glass with SiO_2_ reduces *E_c_* down to 264 kJ mol^−1^ [20] or 199 ± 22 kJ mol^−1^ for Li_1.5_Al_0.5_Ge_1.5_P_2.5_Si_0.5_O_12_ glass [21], while the lithium-ion conductivity of the glass-ceramics crystallized at 750 °C is 2.45·10^−4^ S cm^−1^ at RT [22]. Partial substitution of P^5+^ ions by Si^4+^ should result in the formation of sites for Li^+^ ions, which is expected to improve the electrical properties of NASICON-structured glass-ceramics. However, systematic studies of the thermal and structural properties of glasses in the Li_2_O–Al_2_O_3_–GeO_2_–SiO_2_–P_2_O_5_ system for further production of Li_1.5+x_Al_0.5_Ge_1.5_Si_x_P_3−x_O_12_ glass-ceramics have not yet been carried out.

In this paper, we report the effects of P_2_O_5_/SiO_2_ substitution on the thermal, electrical, and structural properties of Li_2_O–Al_2_O_3_–GeO_2_–P_2_O_5_ glasses for the creation of a promising solid electrolyte membrane for all-solid state batteries.

## 2. Experimental

Bulk glass samples of the Li_1.5+x_Al_0.5_Ge_1.5_Si_x_P_3−x_O_12_ (*x* = 0, 0.1, 0.2, 0.3, 0.4, 0.5) series were prepared by the standard melt quenching method using Li_2_CO_3_ (>99.4%, Reakhim, Moscow, Russia), Al_2_O_3_ (>99.9%, Reakhim, Moscow, Russia), GeO_2_ (>99.9%, Reakhim, Moscow, Russia), SiO_2_ (>98.0%, Reakhim, Moscow, Russia), and NH_4_H_2_PO_4_ (≥98.0%, Reakhim, Moscow, Russia). Table 1 shows the compositions of Li_1.5+x_Al_0.5_Ge_1.5_Si_x_P_3−x_O_12_ glass samples. The starting components were thoroughly mixed together. The charge was heated stepwise up to 500 °C with exposure at the final temperature for 2 h to remove volatile components. The resulting mixture was melted in a Pt crucible at 1250 °C for 1 h in air. To obtained glasses, the melt was quenched between preheated steel plates with cooling rate ~10^2^ °C min^−1^. Then all obtained samples were annealed at 420–500 °C for 2 h depending on the composition and cooled slowly to RT in a furnace at a rate of 1 °C min^−1^. As a result, transparent colorless parallel-sided plates without any impurities were obtained.

The amorphous structure of the obtained glasses and the crystalline phases present after heat treatment were determined by X-ray diffraction method (XRD) on a Rigaku D/MAX-2200VL/PC diffractometer (Rigaku Corporation, Tokyo, Japan) using Cu Kα radiation in the range of 10 ≤ 2θ ≤ 80 at RT.

The chemical composition of the glasses was determined by atomic emission spectroscopy (AES) with inductively coupled plasma using an Optima 4300 DV (PerkinElmer, Waltham, MA, USA) spectrometer. The measurement accuracy was 2–3%.

The glass transition temperature (*T_g_*), crystallization onset temperature (*T_c_*), and crystallization peak temperature (*T_p_*) were established by differential scanning calorimetry (DSC) on a thermal analyzer Netzsch STA 449 F1 Jupiter (NETZSCH-Gerätebau GmbH, Selb, Germany) at the rate of 3, 5, 10, 15, and 25 °C min^−1^ in Pt crucibles in the 35–750 °C temperature range in air (20 mL min^−1^).

Linear thermal expansion was investigated on the samples in the form of rectangular glass bars in a push-rod quartz dilatometer. The measurements were performed by Tesatronic TT80 (TESA, Urdorf, Switzerland) digital meter with a high-precision TESA GT 21HP probe (a sensitivity of 0.01 μm) in the temperature range of 25–600 °C at a heating rate of 3 °C min^−1^.

The density of the samples was estimated by Archimedes principle at 25 °C in several parallels.

The electrical resistance of the samples was measured by the electrochemical impedance method in a two-probe cell with silver metal electrodes in air. An Ellins P-5X potentiostat/galvanostat (Elins, Chernogolovka, Russia) was used for resistance measurement. For this measurement, the samples were polished and coated with Ga-Ag paste to form the electrodes. The impedance spectra were obtained in the frequency range of 0.025–1000 kHz and the temperature range of 150–300 °C.

Raman spectra were recorded at RT on a Renishaw Ramascope U1000 equipped with a confocal Leica DML microscope (Renishaw, New Mills, UK) operating on a solid-state laser (λ = 532 nm) with a power of 5 mW on the sample. Spectral calibration was performed using the Raman spectrum of silica. The spectral resolution was 1 cm^−1^. The intensities were normalized to the maximum value.

Infrared spectra were obtained using a Fourier-transform infrared spectrophotometer (FT-IR) Tensor 27 Bruker (Bruker Optik GmbH, Ettlingen, Germany) and KBr pellet technique. IR spectra were recorded in the wavenumber range 400–4000 cm^−1^ with a spectral resolution of 0.9 cm^−1^ with 32-fold scanning. Sample powders were mixed with KBr (1:200) and pressed to get a transparent pellet.

## 3. Results and Discussion

### 3.1. Characterization and Thermal Behavior of the Glasses

**Figure 1:**
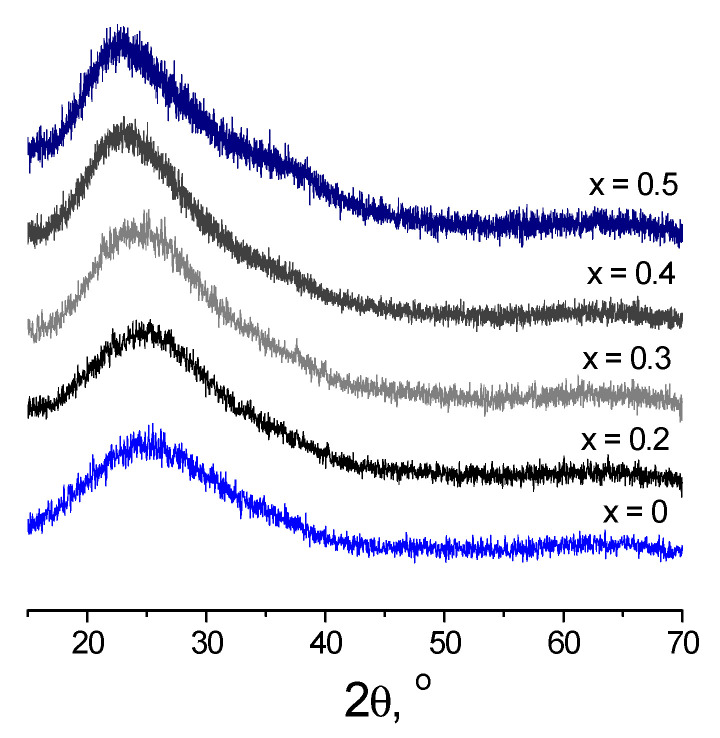
XRD patterns of Li_1.5+x_Al_0.5_Ge_1.5_Si_x_P_3−x_O_12_ glasses.

Figure 1 shows powder diffraction patterns of compositions based on the Li_2_O–Al_2_O_3_–GeO_2_–SiO_2_–P_2_O_5_ system with different additive contents. It can be seen from the XRD data that all samples show haloes characteristic of amorphous materials without peaks of crystalline phases.

**Figure 2:**
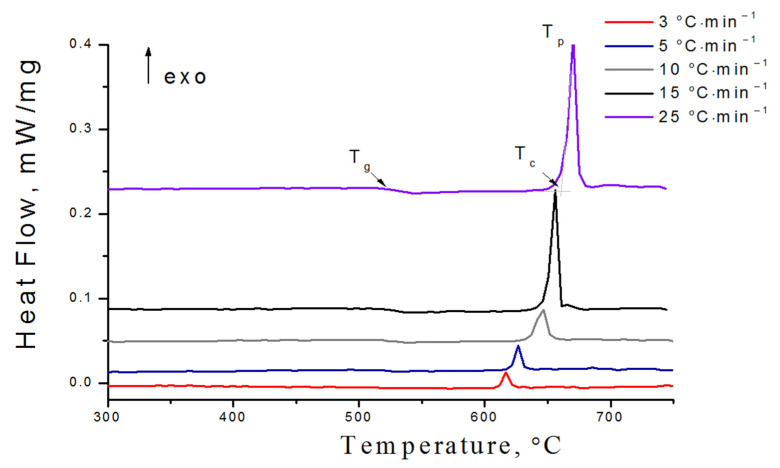
DSC curves of bulk 0.1Si glass at different heating rates.

**Figure 3:**
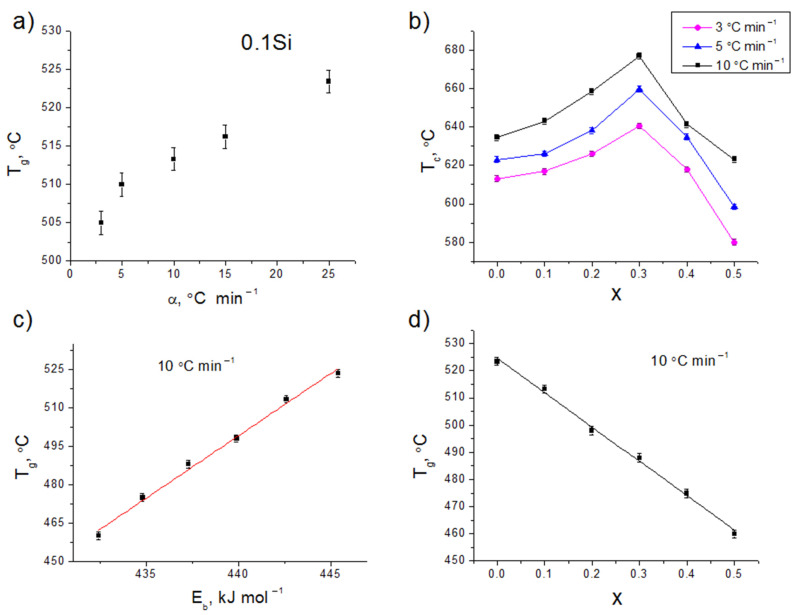
Characteristic temperatures: the glass transition temperatures (*T_g_*) of the cast 0.1Si glass (**a**) and the crystallization peak onset temperatures (*T_c_*) of Li_1.5+x_Al_0.5_Ge_1.5_Si_x_P_3−x_O_12_ glasses at different heating rates (**b**) and at the rate of 10 °C min^−1^ (**c**,**d**).

DSC analysis at different heating rates (3, 5, 10, 15, and 25 °C min^−1^) was performed to understand the crystallization kinetics and thermal stability of glasses. Figure 2 shows the DSC-curves of 0.1Si glass at 10 °C min^−1^. Bends around 500–530 °C depending on the heating rate for 0.1Si glass are related to the glass transition temperature (*T_g_*), while exothermic reactions indicate the crystallization process. As can be seen, *T_g_* increases from 505 °C to 523.4 °C with an increase in the heating rate from 3 to 25 °C min^−1^ (Figure 2 and Figure 3a). On the DSC curves of SiO_2_-contained glasses at a heating rate of 10 °C min^−1^, *T_g_* decreases gradually from 523.4 to 460.0 °C with increasing *x* from 0 to 0.5 (Figure 3d). This is probably due to the substitution of P–O bonds (589 kJ mol^−1^) [23] by Si–O bonds (452 kJ mol^−1^) [24] with a lower bond enthalpy.

The values of *T_g_* correlated with the average single bond enthalpy (*E_B_*) of glasses (Figure 3c), which was calculated as:

(1) $$E_{B}=\frac{x\cdot E_{Li-O}+y\cdot E_{Al-O}+z\cdot E_{Ge-O}+a\cdot E_{Si-O}+b\cdot E_{P-O}}{100}$$

where *x*, *y*, *z*, *a*, *b* are the content of the corresponding oxides in mol%; $E_{Li-O}$, $E_{Al-O}$, $E_{Ge-O}$, and $E_{Ge-O}$ are single bond dissociation energies for Li–O (341 kJ mol^−1^) [25], Al–O (512 kJ mol^−1^) [25], Ge–O (343 kJ mol^−1^) [26], Si–O (452 kJ mol^−1^) [24], and P–O (589 kJ mol^−1^) [23], respectively.

Figure 3c shows the change in *T_g_* depending on the *E_B_* of the compositions. As can be seen, *T_g_* increases with increasing *E_B_*. Similar dependences were also obtained for other oxide glasses [25]. It is well-known that *T_g_* depends on the cross-link density and closeness of the packing of the glass [27,28,29], which will be considered in Section 3.4. Another reason for these changes in *T_g_* is probably in reducing the glass network connectivity as the SiO_2_/P_2_O_5_ ratio increases. It is noteworthy that an increase in the *x* content is accompanied by the increase in the ratio of the dopants (Li_2_O + Al_2_O_3_) to the glass formers (GeO_2_ + SiO_2_ + P_2_O_5_) in the studied series of glasses (Table 1). Modifiers destroy the chains in the glass network, causing a decrease in *T_g_* with increasing *x* (Figure 3d).

**Figure 4:**
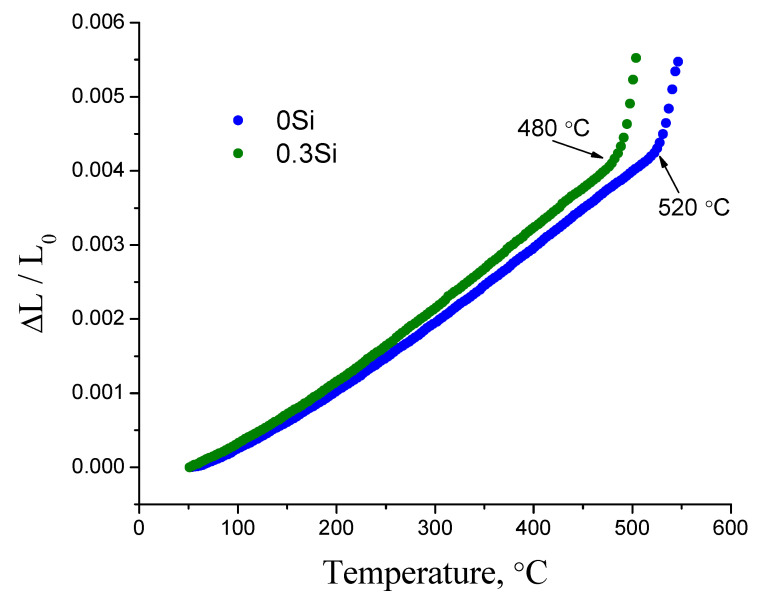
Thermal expansion dependences of 0Si and 0.3Si glasses.

In addition, the glass transition point (*T_g_*) was determined by push-rod quartz dilatometry to compare the results with DSC data. The glass transformation temperature was determined from the change in the slope of the elongation versus temperature plot (Figure 4). The *T_g_* from thermal expansion was found to be 520 °C compared to 519.7 °C for 0Si glass at the same heating rate (3 °C min^−1^). Figure 4 shows that *T_g_* decreases while the thermal expansion coefficient increases with the additive content.

### 3.2. Crystallization Behavior

The crystallization peak onset temperatures (*T_c_*) and the crystallization peak temperatures (*T_p_*) shift toward higher values (Figure 3b, Table 2) as the heating rate increases. A similar behavior is also characteristic of other glassy systems [30]. An increase in *x* is accompanied by a gradual increase in *T_c_* from 623 °C (*x* = 0) to 659.7 °C (*x* = 0.3) followed by a considerable decrease to 598.5 °C (*x* = 0.5) at a constant heating rate (5 °C min^−1^), which should be related to structural changes in the glass network.

The thermal stability of glasses was determined as ∆*T* = *T_c_* − *T_g_* and is given in Table 2 for different heating rates. It has been established that ∆*T* increases from 111.1 °C (*x* = 0) to 188.9 °C (*x* = 0.3), and then decreases to 163 °C (*x* = 0.5) at the rate of 10 °C min^−1^. An extremum in the plot of thermal stability vs. concentration at *x* = 0.3 is observed for all heating rates. An increase in the thermal stability of the glasses up to *x* = 0.3 indicates an increase in the glass formation temperature range to obtain the desired membrane geometry.

The activation energy for crystallization (*E_c_*) of glasses is an important parameter in the analysis of the crystallization process of glasses for the glass-ceramics production. *E_c_* was calculated by the Kissinger equation:

(2) $$\ln(\frac{\alpha }{T_{p}^{2}})=\left(-\frac{E_{c}}{RT_{p}}\right)+const$$

where *R* is the ideal gas constant and α is the heating rate.

**Figure 5:**
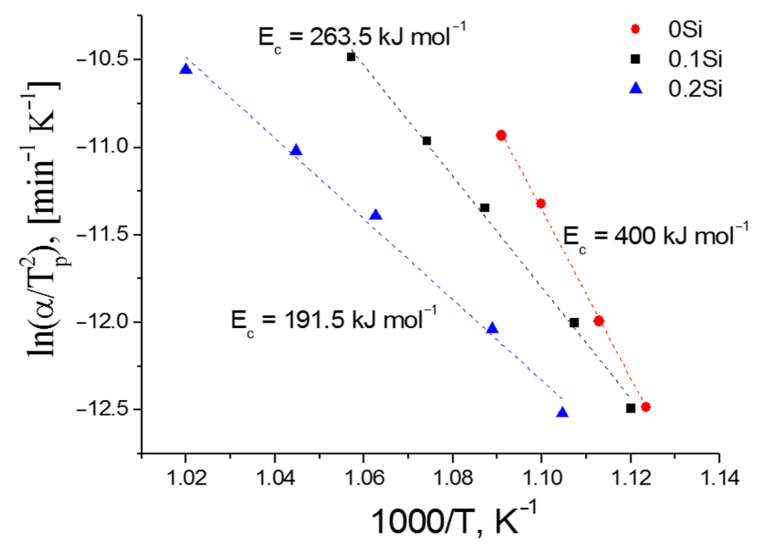
Kissinger plots of $\ln\left(\frac{\alpha }{T_{p}^{2}}\right)$ versus 1/*T_p_* for 0Si–0.2Si glasses.

**Figure 6:**
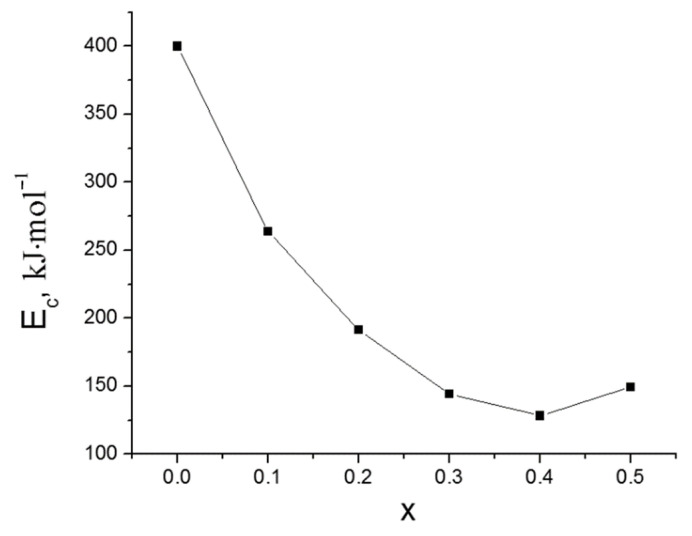
Crystallization activation energy of Li_1.5+x_Al_0.5_Ge_1.5_Si_x_P_3−x_O_12_ glasses as a function of *x*.

Figure 5 shows plots of the dependence $\ln\left(\frac{\alpha }{T_{p}^{2}}\right)$ versus 1/*T_p_* for the glasses obtained. The *E_c_* calculated from the slope of the linear curve shown in Figure 5 is 400 kJ mol^−1^ for 0Si glass (Li_1.5_Al_0.5_Ge_1.5_(PO_4_)_3_ composition) and is in good agreement with the data of [16,31], confirming the correctness of our data. *E_c_* initially decreases with increasing *x* content and reaches a minimum at *x* = 0.4 (Figure 6). A similar trend in *E_c_* with SiO_2_ doping was obtained in [20,21]. The introduction of SiO_2_ was found to decrease *E_c_* down to 128 kJ mol^−1^; therefore, less energy is required for incorporating crystals into the Li_2_O–Al_2_O_3_–GeO_2_–SiO_2_–P_2_O_5_ glass matrix. Hence, a Si-containing glass-ceramic membrane can be obtained at temperatures below 820 °C, which is optimal for obtaining Li_1.5_Al_0.5_Ge_1.5_(PO_4_)_3_ solid electrolyte [11].

The phase composition of the glass-ceramic samples after heat treatment at 820 °C for 2 h was determined. According to XRD data, LiGe_2_(PO_4_)_3_ with a NASICON-type structure is formed together with the impurity phases of AlPO_4_, Li_4_P_2_O_7_, SiO_2_, and Li_9_Al_3_(P_2_O_7_)_3_(PO_4_)_2_, which appear at *x* > 0.1.

### 3.3. Transport Properties of Glasses

**Figure 7:**
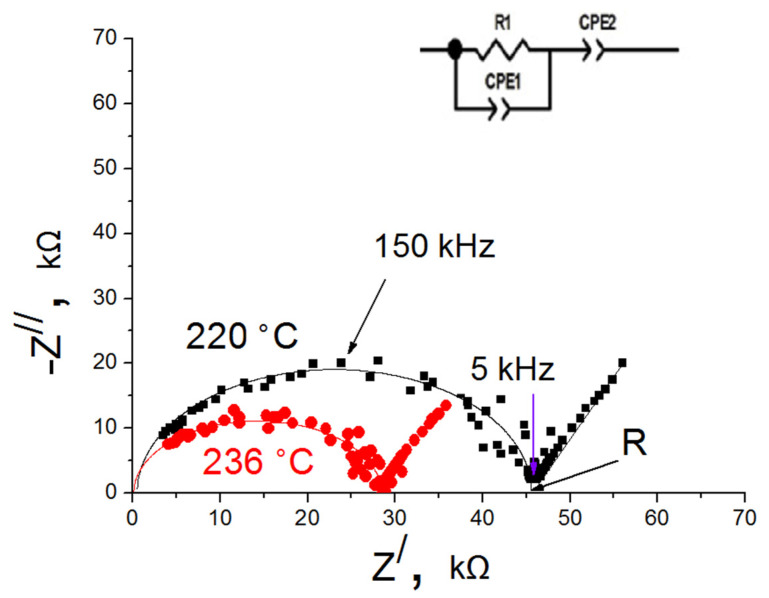
Impedance spectra of 0.3Si glass at 220 °C (black) and 236 °C (red).

Figure 7 shows typical impedance spectra of the glasses obtained. The impedance spectra have a shape characteristic of ion-conducting glasses and are fitted according to the equivalent circuit (Figure 7 inset). A similar equivalent circuit was applied in the works [32,33]. The high-frequency semicircle corresponds to bulk response (R) and the low frequency tail characterized the electrode polarization (an additional constant phase element CPE2) [16,34]. It should be noted that the formation of a single arc emerging from the origin is typical for single-phase systems. An increase in the additive content leads to a decrease in the resistance.

**Figure 8:**
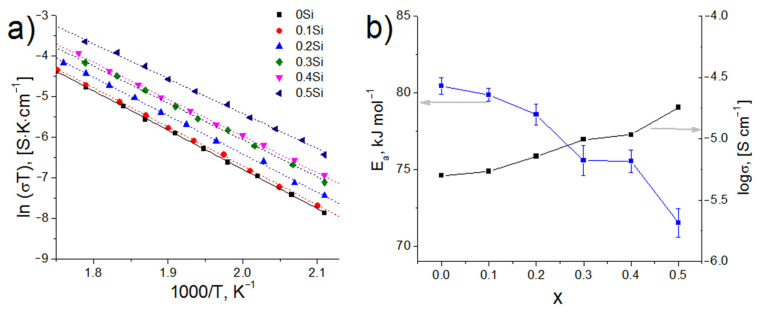
Arrhenius plots of the conductivity for 0Si-0.5Si glasses (**a**) and composition dependences of the *E_a_* (blue) and conductivity (black) of Li_1.5+x_Al_0.5_Ge_1.5_Si_x_P_3−x_O_12_ glasses at 250 °C (**b**).

**Figure 9:**
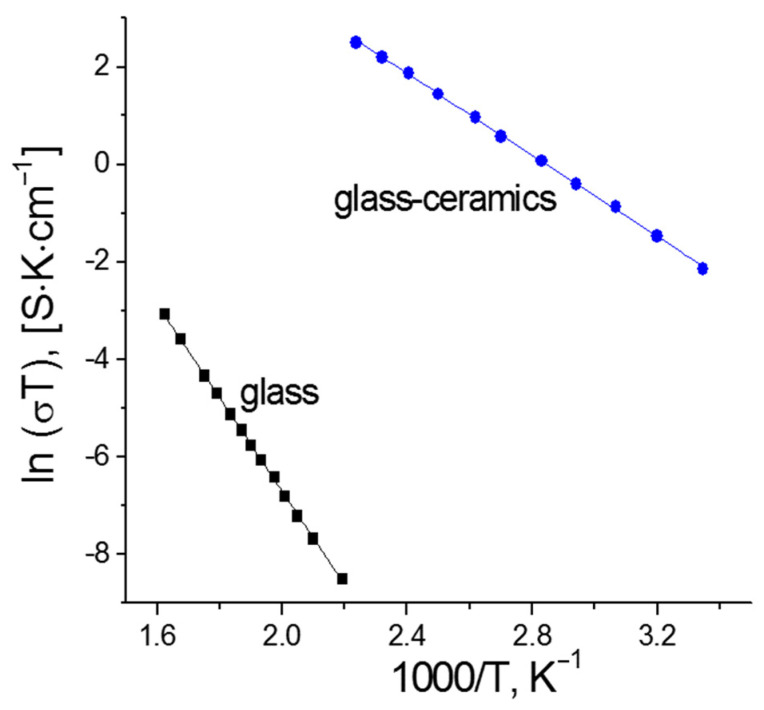
Comparison of total conductivity for Li_1.6_Al_0.5_Ge_1.5_Si_0.1_P_2.9_O_12_ glass and glass-ceramics.

Taking into account the fitted values of the resistance according to the equivalent circuit and the geometry of the samples, the specific conductivity of the glasses (*σ*) was calculated at different temperatures (Figure 8a). It has been found that the conductivity of all compositions demonstrates an Arrhenius temperature dependence, which indicates the absence of phase transitions in the temperature range studied and agrees with the DSC data. According to the Arrhenius equation [16], the activation energy for conduction (*E_a_*) was calculated from the temperature dependences of conductivity. *E_a_* decreases from 80.4 ± 0.5 kJ mol^−1^ (0.83 ± 0.01 eV) to 71.5 ± 0.9 (0.74 ± 0.01 eV) kJ mol^−1^ for *x* = 0 and *x* = 0.5, respectively, as the conductivity increases (Figure 8b). The electrical conductivity of the parent glasses for glass-ceramics production at room temperature is <10^−10^ S·cm^−1^, however, heat treatment of these glasses under optimal conditions increased the conductivity by several orders of magnitude up to ~10^−4^ S·cm^−1^ (Figure 8b and Figure 9).

### 3.4. Short-Range Structure of the Glasses

**Figure 10:**
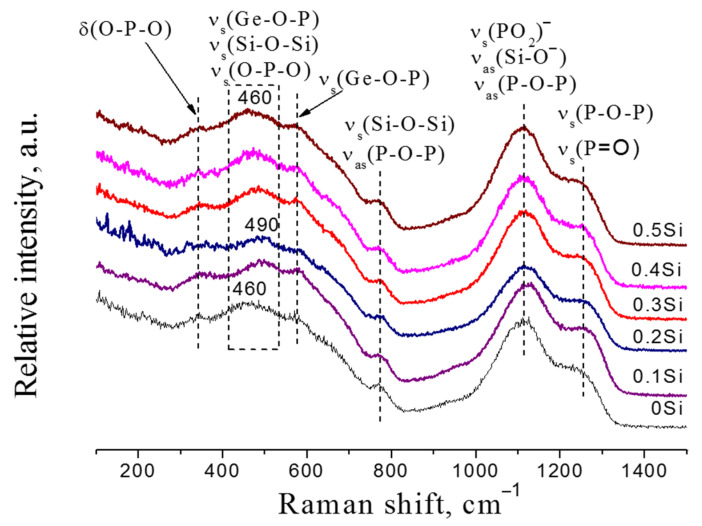
Raman spectra for 0Si–0.5Si glasses at room temperature.

The changes in the crystallization behavior, thermal and transport properties of the glasses investigated due to short-range structural changes were studied by Raman and IR spectroscopy. Figure 10 shows the evolution of the Raman spectra with *x* content.

The multicomponent glasses under study contain PO_4_, GeO_4_, and SiO_4_ tetrahedra, which form various types of connections between themselves and groups with bridging and non-bridging oxygen atoms. At the stoichiometric ratio O/P = 4, orthophosphate groups (Q^1^) should prevail in the glass network, which was pointed out in [35].

The Raman bands near 600–1400 cm^−1^ are due to phosphate units, and the bands at 400–1000 cm^−1^ range are due to germanate units introduced into phosphate chains. The correlation of bands with vibration modes is given in Table 3. The shoulder around 1255 cm^−1^ is related to the P=O vibrations [36,37,38] and the symmetric stretching vibrations of the P–O–P bond [39]. The peak at 1115 cm^−1^ is associated with the asymmetric stretching vibrations of the P–O–P bond [39,40] and symmetric stretching vibrations of the Q^2^ phosphate tetrahedra [36,37,38,39]. In addition, this band indicates the formation of non-bridging oxygen associated with Q^3^ SiO_4_ tetrahedra [40,41,42]. The band at 775 cm^−1^ is due to the symmetric and asymmetric stretching vibrations of the P–O–P bond [37,38,39,40,42] and symmetric stretching vibrations of the Si–O–Si bond [42,43]. The bands around 460 and 575 cm^−1^ are related to symmetric stretching vibrations of the Ge–O–P bond [11] and also the vibrations of the phosphate and silicate tetrahedra [42,44], respectively. With increasing *x* content, the most intense band at 460 cm^−1^ shifts to 490 cm^−1^ for *x* = 0 and *x* = 0.2, respectively. Then the band at 490 cm^−1^ moves to 460 cm^−1^ up to *x* = 0.5, while some bands remain unchanged. This should be due to the destruction of Ge–O–P bonds and the appearance of new Ge–O–Si or Ge–O–Ge bonds [11,45,46]. The Raman spectra are difficult to interpret due to the overlap of the bands related to phosphate and silicate units. Additional information about the molecular structure of the glasses under study was obtained using IR-spectroscopy.

The IR-spectra of undoped and SiO_2_-doped glasses are shown in Figure 10. All IR-spectra consist of five relatively broad bands, which indicate a strong modification of the glass network [16]. The bands appearing in the 1100–1200 cm^−1^ region are associated with the vibrations of terminal (Q^1^) phosphate tetrahedra, namely the O–P–O asymmetric stretching vibrations [16,47,48] and asymmetric stretching vibrations of the P–O^-^ bond [36,37,49]. The shoulder at around 950 cm^−1^ results from the asymmetric stretching vibrations of both P–O–P and Ge–O–Ge bonds [16,50,51,52]. The band centered at 775 cm^−1^ is due to symmetric Ge–O–P or P–O–P stretching vibrations [16,50,52]. The shoulder around 1260 cm^−1^, related to the P=O vibrations [50,53], is very weak because stronger P–O–Ge or P–O–Si bonds are formed.

As the *x* content increases, several main features are observed: (i) the band at 958 cm^−1^ (*x* = 0) shifts to 940 cm^−1^ (*x* = 0.5), (ii) the intensity of the band at 775 cm^−1^ becomes smaller up to *x* = 0.4, (iii) the 510 cm^−1^ band moves toward a lower wavenumber reaching 490 cm^−1^ in the spectrum of *x* = 0.2, and then shifts to 507 cm^−1^ for *x* = 0.5. These changes indicate the gradual depolymerization of the phosphate network with the formation of a mixed complex silicon-phosphate-germanate glass network, which results in a decrease in the density of the samples (Table 1). The loosening of the glass network is due to the growing number of the modifiers (Li_2_O + Al_2_O_3_) and the decrease in the number of the glass-formers (GeO_2_ + SiO_2_ + P_2_O_5_).

**Figure 11:**
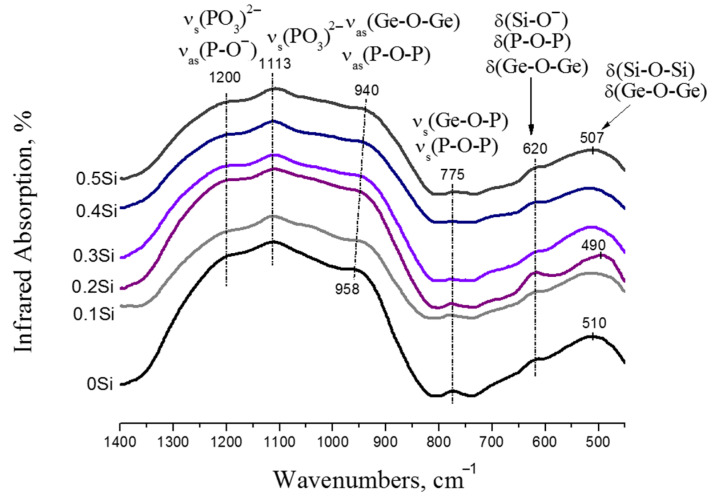
FT-IR spectra of 0Si–0.5Si glasses.

The decrease of *T_g_* and the increase of the thermal expansion coefficient may be related to the loosening of the glass network, i.e., to the growing number of Q^1^ phosphate units. As can be seen from Figure 11, the IR-spectra of 0Si and 0.1Si compositions, as well as those for 0.3Si and 0.4Si compositions, have a similar appearance and, as can be seen from Figure 8a,b, their conductivity values are close. The growth of lithium-ion conductivity of SiO_2_-doped glasses is due to two factors: an increase in the number of non-bridging oxygen atoms, which are sites for the migration of Li^+^ ions, and the increase in the concentration of charge carriers (Li^+^).

## 4. Conclusions

The effect of P_2_O_5_/SiO_2_ substitution on the Li_2_O–Al_2_O_3_–GeO_2_–P_2_O_5_ glasses examined by DSC shows that *T_g_* decreases from 523.4 to 460 °C as the *x* content increases from 0 to 0.5, respectively, due to the substitution of P–O bonds (589 kJ mol^−1^) for Si–O (452 kJ mol^−1^) with the lower bond enthalpy. The change in *T_g_* is consistent with the results of dilatometry. A correlation between *T_g_* and *E_B_* was established. It was found that the thermal stability of glasses increases up to *x* = 0.3, which indicates the increase in the temperature range for the formation of SiO_2_-containing glasses in order to obtain the desired membrane geometry. The activation energy of glass crystallization significantly decreases from 400 to 128 kJ mol^−1^ for *x* = 0 and *x* = 0.4, respectively. Thus, the Si-containing glass-ceramic membrane can be obtained at temperatures below 820 °C, which is optimal for obtaining SiO_2_-undoped glass-ceramics. The Li^+^ conductivity of the glasses increases as a function of *x*. The changes in the thermal and electrical properties with the change in the content of *x* are related to short-range structural changes in the glasses. The infrared spectra show the formation of the Q^1^ phosphate groups as *x* increases. The results of structural studies demonstrate the gradual depolymerization of the phosphate network. So, the decrease in the connectivity of the glass network, which accompanies the increase in SiO_2_/P_2_O_5_ ratio, is the reason for the decrease in *T_g_* and the enhancement in conductivity. It should be noted that the conductivity of the glass-ceramics obtained from SiO_2_-doped glasses has high values (>10^−4^ S cm^−1^ at RT). Therefore, they can be considered as promising solid electrolytes for all-solid-state batteries.

## Figures and Tables

**Table 1:** Compositions of Li_1.5+x_Al_0.5_Ge_1.5_Si_x_P_3−x_O_12_ glasses in mole percent and sample density (ρ).

Glass Code	*x* Value	Method	Li_2_O, mol%	Al_2_O_3_, mol%	GeO_2_, mol%	SiO_2_, mol%	P_2_O_5_, mol%	(Li+Al)/ (Ge+Si+P)	O/P	ρ ± 0.04, g cm^−3^
0Si	0.0	nominal	18.75	6.25	37.50	–	37.50	0.33	4.1	2.98
		AES	18.0	7.0	38.1	–	36.9			
0.1Si	0.1	nominal	19.51	6.09	36.59	2.44	35.37	0.34	4.2	3.03
		AES	18.9	6.9	37.0	2.5	34.7			
0.2Si	0.2	nominal	20.24	5.95	35.71	4.76	33.34	0.35	4.3	3.01
		AES	20.1	6.0	36.2	4.6	33.1			
0.3Si	0.3	nominal	20.93	5.81	34.88	6.98	31.40	0.36	4.5	3.05
		AES	20.8	5.9	35.3	7.1	30.9			
0.4Si	0.4	nominal	21.59	5.68	34.09	9.09	29.55	0.36	4.6	2.96
		AES	20.9	5.6	34.3	9.0	30.2			
0.5Si	0.5	nominal	22.22	5.56	33.33	11.11	27.78	0.38	4.7	2.87
		AES	22.0	5.6	33.0	11.1	28.3			

**Table 2:** The values of characteristic temperature of Li_1.5+x_Al_0.5_Ge_1.5_Si_x_P_3−x_O_12_ glasses: glass transition temperatures (*T_g_*), crystallization peak onset temperatures (*T_c_*), crystallization peak temperatures (*T_c_*) and thermal stability (∆*T*) at different heating rates (*α*). The measurement accuracy of the characteristic temperatures was ±1.5 °C.

x	α, °C min^−1^	*T_g_*, °C	*T_c_*, °C	*T_p_*, °C	∆*T*, °C
0	3	519.7	613	616.9	93.3
	5	523	623	625.4	100
	10	523.4	634.5	636.0	111.1
0.1	3	505	616.9	619.6	111.9
	5	510	626.1	629.9	116.1
	10	513.3	642.9	646.6	129.6
	15	527.6	652.9	657.8	125.3
	25	523.4	665.2	672.8	141.8
0.2	3	495.6	626	632.1	130.4
	5	498.4	638.1	645.3	139.7
	10	498	658.5	667.9	160.5
0.3	3	472	640.5	646	168.5
	5	485.1	659.7	667.1	174.6
	10	488	676.9	701.9	188.9
0.4	3	458.7	617.8	631.9	159.1
	5	475.7	634.8	655	159.1
	10	474.9	641.2	692.5	166.3
0.5	3	440	580	631.2	140
	5	462	598.5	653.7	136.5
	10	460	623	683.8	163

**Table 3:** Assignments of various vibrational bands from Raman spectra of the glasses obtained.

Band Position, cm^−1^	Band Assignments	References
1255	ν_s_ (P–O–P)	[39]
	ν_s_ (P=O)	[36,37,38]
1115	ν_s_ (PO_2_)^−^	[36,37,38,39]
	ν_as_ (P–O–P)	[39,40]
	ν_as_ (Si–O^−^)	[40,41,42]
775	ν_as_ (P–O–P)	[38,39,40]
	ν_s_ (P–O–P)	[37,42]
	ν_s_ (Si–O–Si)	[42,43]
575	ν_s_ (O–Si–O)	[42,44]
	ν_s_ (O–P–O)	[42]
	ν_s_ (Ge–O–P)	[11]
460–490	ν_s_ (O–P–O)	[41,42]
	ν_s_ (Ge–O–Ge)	[11,45,46]
340	δ (O–P–O)	[37]

## Data Availability

Not applicable.
